# Atypical brain lateralization for speech processing at the sublexical level in autistic children revealed by fNIRS

**DOI:** 10.1038/s41598-024-53128-7

**Published:** 2024-02-02

**Authors:** Baojun Lai, Aiwen Yi, Fen Zhang, Suiping Wang, Jing Xin, Suping Li, Luodi Yu

**Affiliations:** 1 Center for Autism Research, School of Education, Guangzhou University, Guangzhou, China; 2 Philosophy and Social Science Laboratory of Reading and Development in Children and Adolescents (South China Normal University), Ministry of Education, Guangzhou, China; 3 Tiyudong Road Primary School (Xingguo), Guangzhou, China; 4 Department of Obstetrics and Gynecology, Department of Pediatrics; Guangdong Provincial Key Laboratory of Major 0bstetric Diseases; Guangdong Provincial Clinical Research Center for Obstetrics and Gynecology; Guangdong-Hong Kong-Macao Greater Bay Area Higher Education Laboratory of Maternal-Fetal Joint Medicine, The Third Affiliated Hospital of Guangzhou Medical University, Guangzhou, China; 5 VITO Health, Flemish Institute for Technological Research, Mol, Belgium; 6 Foshan Clinical Medical School, Guangzhou University of Chinese Medicine, Foshan, China

**Keywords:** Language, Psychology

## Abstract

Autistic children often exhibit atypical brain lateralization of language processing, but it is unclear what aspects of language contribute to this phenomenon. This study employed functional near-infrared spectroscopy to measure hemispheric lateralization by estimating hemodynamic responses associated with processing linguistic and non-linguistic auditory stimuli. The study involved a group of autistic children (*N* = 20, mean age = 5.8 years) and a comparison group of nonautistic peers (*N* = 20, mean age = 6.5 years). The children were presented with stimuli with systematically decreasing linguistic relevance: naturalistic native speech, meaningless native speech with scrambled word order, nonnative speech, and music. The results revealed that both groups showed left lateralization in the temporal lobe when listening to naturalistic native speech. However, the distinction emerged between autism and nonautistic in terms of processing the linguistic hierarchy. Specifically, the nonautistic comparison group demonstrated a systematic reduction in left lateralization as linguistic relevance decreased. In contrast, the autism group displayed no such pattern and showed no lateralization when listening to scrambled native speech accompanied by enhanced response in the right hemisphere. These results provide evidence of atypical neural specialization for spoken language in preschool- and school-age autistic children and shed new light on the underlying linguistic correlates contributing to such atypicality at the sublexical level.

## Introduction

Individuals with autism spectrum disorders (henceforth autism) exhibit atypical language profile. According to the diagnostic criteria outlined in the DSM-5^1^, autistic individuals universally manifest challenges in the social-pragmatic aspect of language. Despite the pronounced heterogeneity in language profiles associated with autism, a majority of children diagnosed with autism concurrently present language disorders, exhibiting delays and difficulties across various core language domains, including phonology, semantics, and syntax^2,3^. The prevalence of co-occurring language disorders is estimated to range from 50 to 60% within the spectrum^4–6^. Approximately 25~30% of autistic children remain nonverbal or minimally verbal^7–10^. One possible neurobehavioral mechanism underlying the language atypicalities in autism is reduced neural specialization for the processing of linguistic structures and vocal signals, as was found in earlier studies on autistic children^11–13^. Left-lateralization of speech processing is a hallmark of specialized language function in the brain^14,15^. This organization has been shown to signify language competence in a variety of populations, including children with typical and atypical language development and second language learners^16–20^. Because autistic children often show subtle or reversed hemispheric lateralization for language processing, understanding the underlying mechanisms is crucial for supporting their language development.

Language-related hemispheric dominance is well-documented in neurotypical infants and children. In neonates, natural speech predominantly activates the left fronto-temporal language regions^21–24^, which is considered to reflect a built-in bias for language learning in the left hemisphere^25^. Research has also established that as children age and their language skills progress, the lateralization of language processing becomes more pronounced^14,26,27^. Such developmental change is thought to be consequential from the growth of individual language experience. Specifically, as age increases and phonological knowledge consolidates, the processing functions of the left and right hemispheres differentiate for various linguistic features and components. Native phonological and linguistic information (e.g., phonemic and semantic processing) tends to be lateralized to the left hemisphere, and nonnative and paralinguistic information (e.g., intonational prosody) becomes specialized within the right hemisphere. Support for the experience-dependent language specialization also comes from adult studies, wherein the successful acquisition of a linguistic form, such as a nonnative language or sinewave speech, is associated with emergence of left lateralization^16,19^.

However, lateralization of the fronto-temporal language regions was found to be substantially deviated in infants and young children diagnosed with autism. Compared to age-matched typically developing (TD) peers, autistic children aged 1–4 years showed lower responsivity in the left superior temporal gyrus (STG) and higher responsivity in the right homologue region when listening to natural speech when asleep^28,29^. It was further demonstrated that this group difference became more prominent at 3- and 4-year of age^29^. Reversed lateralization has also been reported in electrophysiological studies examining cortical processing of words. Specifically, a link between reduced left lateralization in the ERP responses to word meaning and greater autism symptoms was found in 2-year-old autistic toddlers^30,31^.

Atypical lateralization is not a phenomenon confined to early development but also frequently seen in school-age children, adolescents and adults with autism during a variety of language-based tasks, including word production^32^, word detection^33^, sentence comprehension^34–36^, and communicative intent comprehension^35^. Others have found an association between individual language competence and hemispheric lateralization in this population. For example, left temporal lobe activation and language skills in 11- to 16-year-old autistic adolescents were positively correlated, although atypical right frontal activation was also found to be associated with better language abilities as well^37^. In another study, autistic adults who had a history of language delay showed no significant leftward asymmetry in the N400 component, a neurophysiological marker associated with semantic processing, unlike those without a history of language delay^38^. These findings suggest a long-lasting effect of atypical hemispheric specialization that could be an important neural marker for understanding autistic language^12^.

Notably, reduced leftward asymmetry in autism is often accompanied by increased involvement of the right hemisphere^12,39^. In neurotypical brain, the right hemisphere is considered responsible for the processing of paralinguistic and contextual information instead of phonological and lexical processing^14,40,41^. In autistic children who show reduced or reversed lateralization, the right-hemisphere network may compensate for the insufficient development of a language network in the left hemisphere^12,29,37,42^. Enhanced involvement of the right hemisphere in autism has been linked with auditory biases for computational properties of the right hemisphere, namely preferences for spectral information and slow-changing acoustic patterns^13,43,44^. The hyperactivity of the right hemisphere, whether due to compensation or auditory bias, could potentially suppress language functions related to the processing of paralinguistic cues and contextual information, which are typically handled by the right hemisphere. This possibility is in line with the clinical characterization of autism^1^ and lab-based studies that have identified marked difficulties with receptive and expressive prosody, pragmatic and contextual understanding in autistic individuals^45–50^.

Neuroimaging research on language lateralization in autism has mostly utilized higher-order tasks involving multiple linguistic aspects and skills beyond the language network in the brain (e.g., executive function), rendering it difficult to attribute the lateralization differences to specific language factors^35^. Studies with young autistic children using advanced imaging techniques (e.g., functional magnetic resonance imaging or fMRI) often struggle with testability^51^, and have mostly employed native speech without exploring the interaction between hemisphere and speech type^28,29^. Therefore, there remains a knowledge gap regarding the specific aspect within the linguistic hierarchy responsible for the altered lateralization and heightened right hemisphere activities in autism. Addressing this question can offer important information to the developmental mechanisms of neural specialization for language in autistic children with language difficulties.

Functional near-infrared spectroscopy (fNIRS) is a noninvasive neuroimaging technique that utilizes the absorption of near-infrared light to measure the fluctuation of hemoglobin concentrations in the cortex as a proxy for neuronal activity^52,53^. fNIRS has several advantages over other neuroimaging techniques including portability, ability to withstand head movement, affordability, and efficacy in natural experimental setting. fNIRS has been shown to be an effective technique for studying brain function in autism^51,54^ and language development^55,56^. Compared to the enclosed space typical of fMRI research, the bigger visible room with attenuated ambient noise in fNIRS testing provides a more comfortable setting for participants with special needs such as individuals with noise intolerance.

The present study aimed to identify the specific linguistic or phonological component that drives the atypical hemispheric lateralization in autistic children. fNIRS was utilized to examine cortical activation in the bilateral language-related areas when processing auditory signals that varied along the linguistic hierarchy. The linguistic hierarchy was created using four stimulus conditions with parametrically decreasing linguistic relevance or content: naturalistic native speech, native speech with scrambled word order, nonnative speech, and music. We hypothesized that leftward asymmetry in the nonautistic comparison children would decrease parametrically as a function of linguistic relevance of the stimuli. In contrast, autistic children would exhibit atypical patterns of lateralization. The level of linguistic relevance at which the atypical lateralization became evident would provide insight into the specific language component that drives atypical auditory-linguistic processing in autism.

## Methods

### Participants

Thirty autistic children and 25 nonautistic comparison children aged 3–10 years old participated in the study. The children were recruited through advertisements requesting study volunteers in the pediatric department of a local hospital. One child's data were excluded due to left-handedness^57^. Two children's data were excluded due to incompletion of the fNIRS experiment. After preprocessing of fNIRS data, 9 autistic children and three nonautistic children were excluded from final analysis due to channel noise or poor data quality (see fNIRS data processing). Participant attrition details, aligning with reporting guidelines to address phenotypic bias in neuroscientific research^58–60^, are provided in Supplementary Material Table S1. The final dataset consisted of 20 autistic children (1 girl and 19 boys, *M* = 5.8, *SD* = 2.2, 3.7–10.8 years) and 20 nonautistic children (10 girls and 10 boys, *M* = 6.5, *SD* = 1.7, 3.8–9.9 years). There was no significant difference in age between the two groups (*t*_(38)_ =  − 1.12, *p* = 0.269; Table 1).

**Table 1:** Sample characteristics of the autism and the comparison groups.

	Autism	Comparison	*p*
Age	5.8 ± 2.2	6.5 ± 1.7	0.269
PPVT-R score	83.7 ± 14.0	135.1 ± 23.5	< .001
SRS score	70.4 ± 22.6	37.3 ± 17.7	< .001
CARS score	33.5 ± 3.4	-	-
ABC score	63.4 ± 18.4	-	-

*PPVT-R* Age-based standards scores of Peabody Picture Vocabulary Test-Revised, *SRS* Social Response Scale, *CARS* Childhood Autism Rating Scale, *ABC* Autism Behavior Checklist.

The children in the autism group received best-estimate diagnosis of autism by pediatricians with at least 10 years of expertise in diagnosing autism. Because the Autism Diagnostic Observation Schedule (ADOS) and the Autism Diagnostic Interview-Revised (ADI-R) have not been validated or widely adopted in China^61–63^, the autistic participants’ diagnoses were supplemented by Childhood Autism Rating Scale (CARS)^64^ and Autism Behavior Checklist (ABC)^65^. Both are widely used diagnostic instruments in China^62,66^. All 20 children in the autism group had CARS scores at or above the cutoff of 30 for autism (*M* = 33.5, *SD* = 3.4, range = 30–44), and 17 had ABC scores at or above the cutoff of 53 for autism (*M* = 63.4, *SD* = 18.4, range = 15–96). Additionally, Social Response Scale (SRS)^67^ scores were available for 16 autistic children (*M* = 70.4, *SD* = 22.6, range = 36–112) and 12 nonautistic children (*M* = 37.3, *SD* = 17.7, range = 14–83). The autistic children scored significantly higher than the nonautistic children on SRS (*t*_(26)_ = 4.19, *p* < 0.001), indicating greater social communication atypicalities. None of the children had any known genetic condition, psychiatric condition, or neurodevelopmental disorder other than autism. None were regularly taking medications at the time of the study. All the participants spoke Mandarin Chinese as their native language.

Chinese version of the Peabody Picture Vocabulary Test-Revised (PPVT-R)^68,69^ was administered by a trained experimenter to measure the children’s receptive vocabulary. PPVT scores were available for 12 children in the autism group, with 8 children either not meeting the minimal scoring requirement or discontinuing the test due to agitation. The autism group's average PPVT-R score was significantly lower than that of the comparison group (*t*_(30)_ =  − 7.23, *p* < 0.001), indicating lower receptive language level.

### Stimuli

There were four stimulus conditions with decreasing linguistic relevance: natural native speech, native speech with scrambled word order, nonnative speech, and music. In the natural native speech condition (Native), a children’s story “The Mower and the Wolf” was read by a female speaker in Mandarin Chinese, and was expected to produce canonical leftward asymmetry of brain activation in native listeners. In the scrambled native speech condition (Native-scrambled), words within each sentence of the native story were shuffled such that the sentence preserved the phonological and prosodic features of the native speech but eliminated the lexico-semantic content. The nonnative speech (Nonnative) was the original story read in Russian, which allowed testing of language-general phonetic processes. The speech stimuli were recorded by the same female speaker who was a Chinese-Russian bilingual. The music condition (Mozart’s Piano Sonata No. 4 in E flat major) was used as a non-verbal auditory control. Music shares similar acoustic and structural complexity with speech but requires specific tonal pitch processing, which is known to engage distinct brain networks with a rightward asymmetry^70,71^.

**Figure 1:**
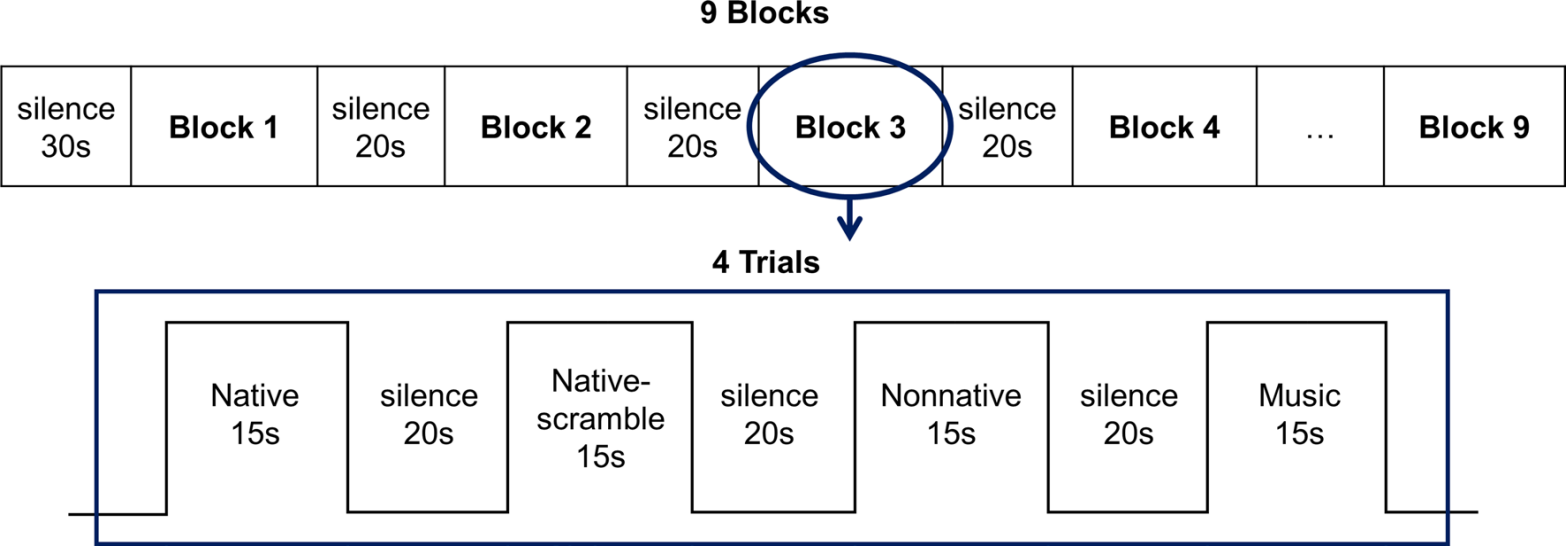
Block design of the current study. The experimental design consisted of 9 blocks, each containing four trials. Individual trials started with a 20-s silence interval, succeeded by a 15-s stimulus segment. Trial presentation within each block occurred in a randomized manner.

Each audio recording was divided into nine 15-s segments (trials; *M* = 15.3 s, *SD* = 0.6 s), resulting in a total of 36 trials. Each speech trial contained complete sentence(s) and each music trial contained entire musical phrases with natural beginnings and endings. A block design was used based on previous studies^23,72^. Each block presented four trials from each of the four conditions in a random order (Fig. 1). There was a 30-s silence before the presentation of stimuli and a 20-s silence between each trial to allow the hemodynamic activity to reset to baseline^55,73^. There were 9 blocks with a total of 21 min.

### Apparatus and procedure

**Figure 2:**
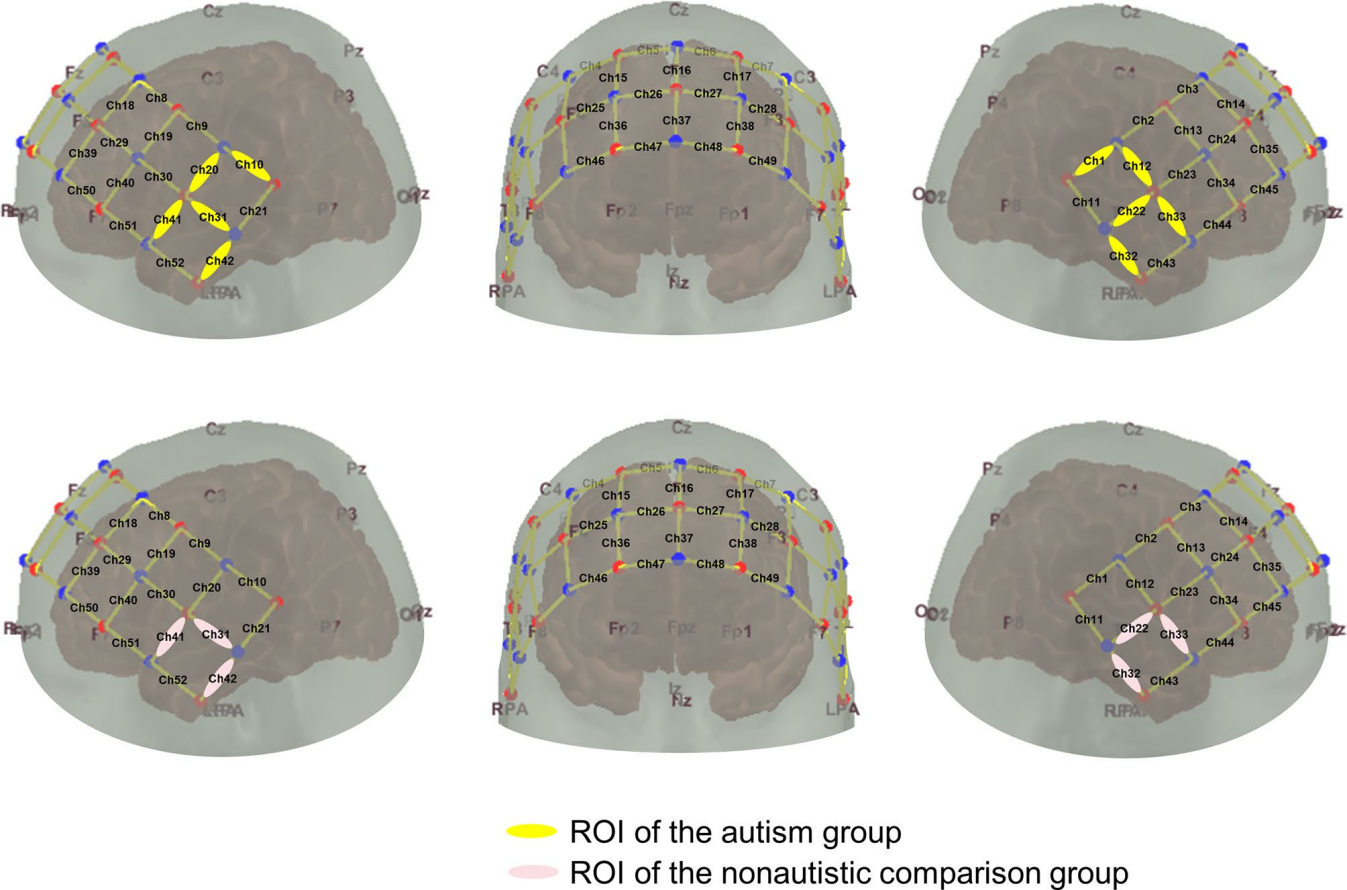
Probeset distribution and channel clusters with significant activation in response to natural native speech (Native). Red circles are the sources, blue circles are the detectors, and thin lines represent channels. The yellow color (5 channels per hemisphere) represent the ROIs in the autism group, and the pink color (3 channels per hemisphere) represent the ROIs in the comparison group.

FNIRS data were collected using a 52-channel continuous wave system (ETG-4000, Hitachi Medical Corporation, Tokyo, Japan) at a sampling rate of 10 Hz (wavelengths: 695 and 830 nm). A 3 × 11 probe set including 17 light sources and 16 detectors was adjusted with elastic straps over the participant's head. The positioning of the optodes was based on the international 10–20 system. To cover the regions of interest, the patch was placed 3–5 cm vertically from the eyebrow centering the middle probe of the most inferior row over Fpz, and the left and right sides were respectively centered at T7 and T8, so that measurement channels covered large areas of temporal and frontal parietal lobe cortex (Fig. 2).

The children were accompanied by their caregivers in a quiet room and sat in a comfortable chair in front of a mobile phone screen used to play cartoons of their own choice. A research assistant positioned the fNIRS headband over designated regions of interest, and made adjustment to ensure the participant’s comfort while maintaining adequate contact between the optodes and the participant’s scalp. The children wore inserted earphones that played the sounds at a comfortable level (70 dB SPL). The children were instructed to watch cartoons while ignoring any sounds from their earphones. They were encouraged to remain still while seated. The child and their parent could withdraw at any time whenever the child expressed discomfort or a desire to stop participation.

The experimental protocol was approved by the Research Ethics Committee of South China Normal University, and all experiments were performed in accordance with relevant guidelines and regulations. The informed consent was obtained from parents or guardians of all participating children in the study.

### fNIRS data processing

The analysis of the fNIRS data was performed using the HOMER2 package^74^ together with custom scripts. Data from the initial 52 participants after preliminary screening (see Participants section) were preprocessed using methods recommended for infant and child data^75,76^. First, the measured light intensity was converted to optical density. Then, channels with optical intensity that were stronger or weaker than the set standard range [mean (d) < 0.001 or > 7] were discarded from the analyses. Next, motion artifacts were defined as signal changes exceeding an amplitude of 0.5 mmol × mm threshold and/or a standard deviation of 50 within a one-second timeframe, to reject the remaining uncorrected motion artifacts of the optical intensity data^77,78^. Wavelet filtering (iqr = 0.8) was performed for de-noising and waveform smoothing^75,79^, and PCA (nSV = 0.85) was performed on detected motion artifacts ^75,80^. The optical density signals were then band-pass filtered to attenuate low-frequency drift and cardiac oscillations with cut-off frequencies of 0.01–0.1 Hz. The signals were converted to estimated changes in the concentration of HbO and HbR through application of the modified Beer-Lambert Law^74^. The hemodynamic responses of trials from a time interval of − 5.0 s to 25.0 s were extracted and averaged for each stimulus condition. After preprocessing, we used plotting and visual inspection to remove noisy channels and trials that were not identified in the previous steps^81–84^. In this step, children were included in the analysis if they had less than or equal to 20 out of 52 channels detected as noisy and less than or equal to 4 out of 36 trials rejected. By applying these criteria, data were available for 20 autistic children and 20 nonautistic children. The average number of trials was 35 per participant.

### Statistical analyses

**Figure 3:**
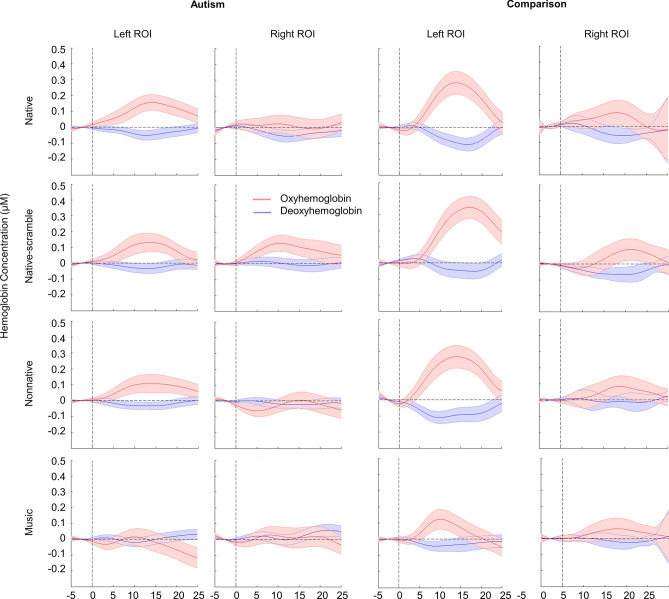
Group-averaged hemodynamic response function (HRF). Red and blue colors represent oxyhemoglobin (HbO) and deoxyhemoglobin (HbR), respectively. Shaded areas indicate standard error values (± 1SE). The significant channel clusters in the left temporal lobe was selected as the left ROI, and the significant channel clusters in the right temporal lobe (symmetrical to the left cluster) was used as the right ROI. The average hemoglobin concentration levels of the channels in ROIs was calculated to obtain the HRF for the autism group and the comparison group in the four stimulus conditions: natural native speech condition (Native), scrambled native speech condition (Native-scrambled), nonnative speech (Nonnative), and music (Music). The x axis represented the time from 5 s pre-stimulation to 25 s post-stimulation.

Nonparametric cluster-based permutation tests (CBPT)^85^ were performed to identify channel clusters showing significant responses in each condition. The signal from multiple channels and time points were subjected to 1000 permutations to reduce the error rate. The HbO and HbR concentration values within 5–25 s post-stimulus were compared with zero baseline. The zero baseline was established by linearly fitting the 5 s preceding trial onset. Significant HbO clusters identified by the CBPT in the Native condition were used as regions of interest (ROI) in each hemisphere. These clusters represent the canonical language region that offers a unified criterion to examine how sounds at different linguistic levels activate this region. Because oxyhemoglobin (HbO) has been reported to show higher signal-to-noise ratio than deoxyhemoglobin (HbR) in children^86,87^ and that no significant HbR clusters were identified for the Native condition in either group, we used HbO for further statistical analysis and HbR was only reported in Table 2 and Fig. 3 for descriptive purposes.

**Table 2:** Significant channel clusters identified by the cluster-based permutation tests.

Group	Index	Condition	Hemis	Channels of cluster	Time (s)	*p*	*T-statistic*
Autism	HbO	Native	Left	10, 20, 31, 41, 42	13.3–23.1	.012 *	1080.99
		Native-scramble	Right	11, 12, 13, 22, 24, 25, 33, 35, 45, 46	5.8–21.0	< .001 *	2154.87
			Left	20, 30, 31, 41, 42, 52	5.9–20.1	.003 *	1658.85
		Nonnative	Left	28, 39, 49	19.5–25.0	.049	− 440.06
Comparison	HbO	Native	Left	31, 41, 42	12.6–21.7	.007 *	1147.48
		Nonnative	Left	31, 41, 42	12.8–18.4	.043	566.03
	HbR	Nonnative	Left	30, 31, 41	6.7–12.1	.021 *	− 436.34

Hemis = Hemisphere. HbO = oxyhemoglobin. HbR = deoxyhemoglobin. Four conditions: natural native speech condition (Native), scrambled native speech condition (Native-scrambled), nonnative speech (Nonnative), and music (Music). Two-sided test, * *p* < .025.

Linear mixed modeling (LMM) implemented by the R package lme4^88^ was used to assess the effects and interactions of the factors of interest on HbO. First, we evaluate whether the groups differed in responses in the two hemispheres: *model1* = *lmer (meanOxyhemoglobin* ~ *Age* + Group*Hemisphere + *(1|subject))*. In this model, age was first entered as a controlled variable; group (autistic or nonautistic), hemisphere (left or right), and their interactions were fixed factors, while subject was entered as a random effect to control for individual variance. Additionally, the effects of Hemisphere × Condition were examined for autism and nonautistic separately: *model2* = *lmer (meanOxyhemoglobin* ~ *Age* + Condition*Hemisphere + *(1|subject))*. Finally, Pearson correlation analysis was performed to examine potential brain-behavior relationships between neural responses to different sound classes and clinical measures.

## Results

### Selection of regions of interest

Table 2 shows the significant channel clusters identified by the cluster-based permutation tests for each stimulus condition in reference to baseline.

According to the permutation test results, the autism and comparison groups had overlapping channels showing significant activation in the Native condition (Table 2, Fig. 2). Therefore, in both groups, the set of significant channel clusters in the left temporal lobe was selected as the left ROI, and the set of significant channel clusters in the right temporal lobe (symmetrical to the left cluster) was used as the right ROI (Fig. 2). The mean HbO concentration for each participant within each hemisphere was computed for further statistical analysis. Figure 3 shows the group-averaged hemodynamic response function (HRF) in each hemisphere in each stimulus condition.

### Lateralization patterns in the autism and comparison groups

**Figure 4:**
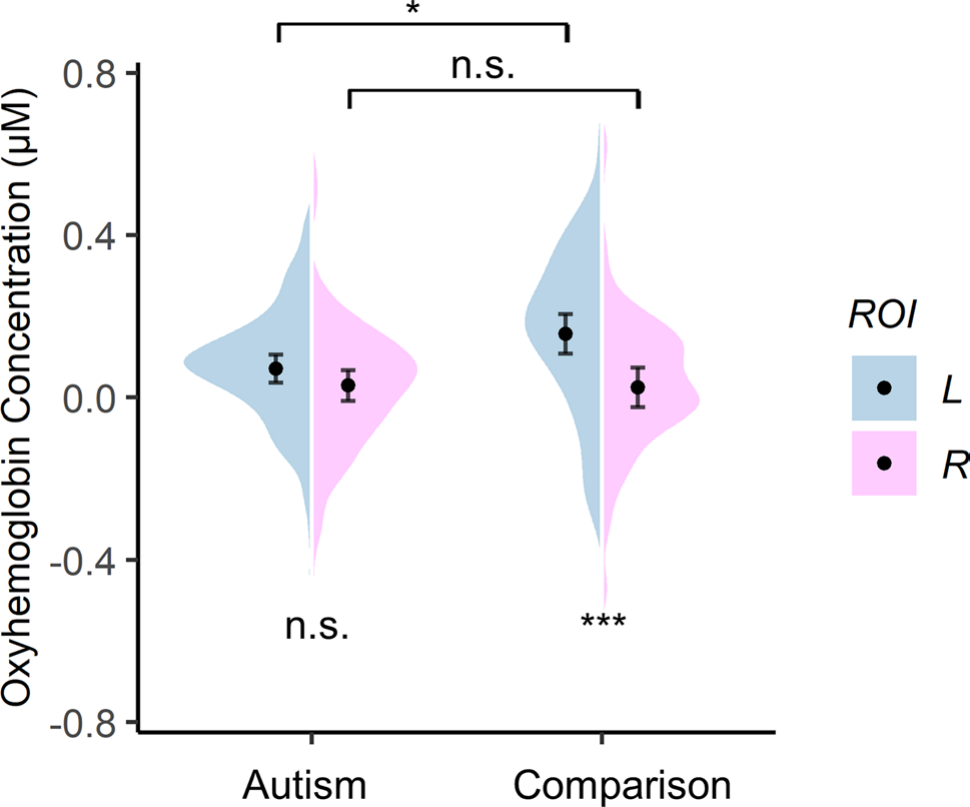
Group × Hemisphere interaction. Overall oxyhemoglobin concentrations in the left ROI (L) and the right ROI (R). Black dots in the violin shapes represent group average levels. Error bars indicate 95% confidence interval. *p < .05, ***p < .001.

In the LME regression combining both groups, there was a significant two-way interaction of Group × Hemisphere (*F* = 5.01, *p* < 0.05, *η*^2^_p_ = 0.01; Fig. 4). Post hoc comparisons indicated that HbO concentration in the left ROI was significantly greater than that in the right ROI in the comparison group (*t*_(278)_ = 4.61, *p* < 0.001, *Cohen’s d* = 0.73, 95% *CI* = [0.41, 1.05]) but not in the autism group (*t*_(278)_ = 1.45, *p* = 0.150, *Cohen’s d* = 0.23, 95% *CI* = [− 0.08, 0.54]); The comparison group had a greater response than the autism group in the left ROI (*t*_(76.7)_ = − 2.60, *p* < 0.05, *Cohen’s d* =  − 0.41, 95% *CI* = [− 0.72, − 0.09]) but response amplitude was comparable between the two groups in the right ROI (*t*_(76.7)_ =  − 0.06, *p* = 0.948, *Cohen’s d* =  − 0.01, 95% *CI* = [− 0.32, 0.30]).

**Figure 5:**
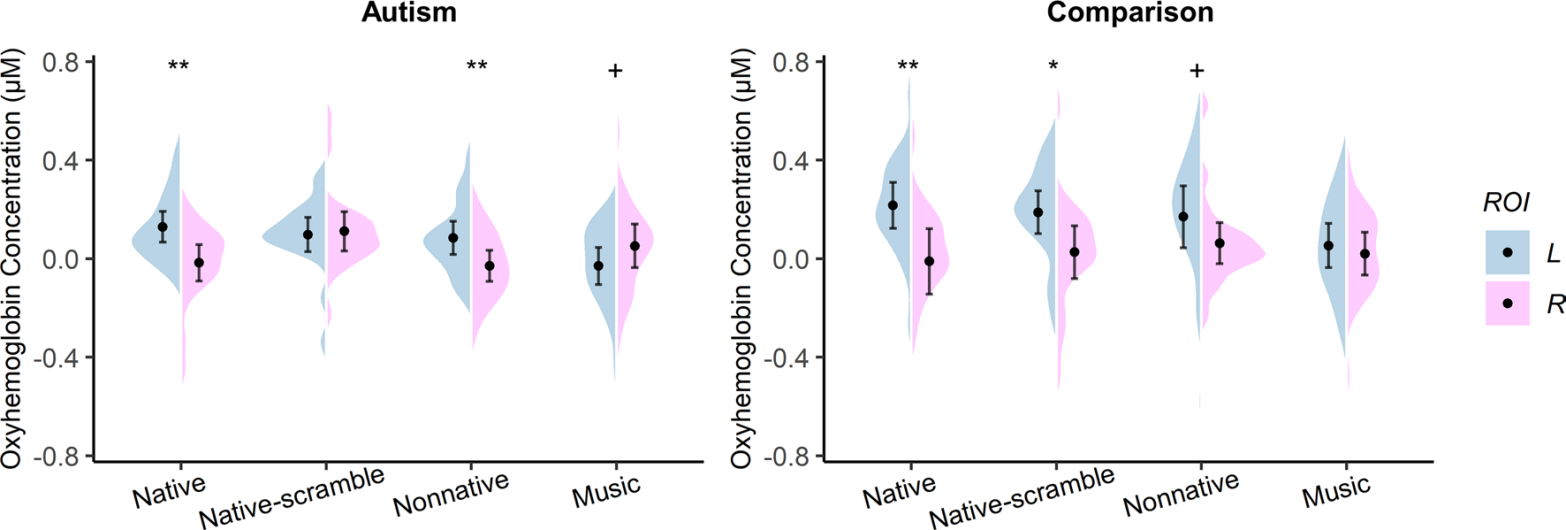
Hemispheric lateralization for the four stimulus conditions in the autism group and the comparison group. Black dots in the violin shapes represent the group means. Error bars indicate 95% confidence interval. *p < .05, **p < .01, + *p* < .1.

We then analyzed the effects of Condition × Hemisphere for each group separately. In the autism group, the Condition × Hemisphere interaction was significant (*F* = 4.87, *p* < 0.01, *η*^2^_p_ = 0.09). Further analysis revealed that left hemisphere activation decreased as linguistic relevance decreased (Native > Native-scramble > Nonnative > Music), and that the right hemisphere was the most responsive for the Native-scrambled speech. In addition, left lateralization was evident in the Native and Nonnative conditions (Native: *t*_(133)_ = 3.02, *p* < 0.01, *Cohen’s d* = 0.96, 95% *CI* = [0.29, 1.61]; Nonnative: *t*_(133)_ = 2.35, *p* = 0.020, *Cohen’s d* = 0.74, 95% *CI* = [0.10, 1.38]). In the Music condition, there was a trend towards right lateralization (*t*_(133)_ = − 1.68, *p* = 0.096, *Cohen’s d* =  − 0.53, 95% *CI* = [− 1.16, 0.10]). The only condition lacking hemispheric difference was the Native-scramble condition (*t*_(133)_ = − 0.27, *p* = 0.785, *Cohen’s d* = − 0.09, 95% *CI* = [− 0.70, 0.54]; Fig. 5).

In the comparison group, no significant Condition × Hemisphere interaction was found. However, exploratory analysis revealed that the effect size of hemispheric difference decreased as a function of linguistic relevance of the stimuli, suggesting decreasing left lateralization (Native: *t*_(133)_ = 3.66, *p* < 0.001, *Cohen’s d* = 1.16, 95% *CI* = [0.48, 1.82]; Native-scramble: *t*_(133)_ = 2.60, *p* = 0.010, *Cohen’s d* = 0.82, 95% *CI* = [0.17, 1.46]; Nonnative: *t*_(133)_ = 1.73, *p* = 0.086, *Cohen’s d* = 0.55, 95% *CI* = [− 0.09, 1.18]; Music:* t*_(133)_ = 0.54, *p* = 0.593, *Cohen’s d* = 0.17, 95% *CI* = [− 0.45, 0.79]; Fig. 5).

### Brain-behavior correlations

**Figure 6:**
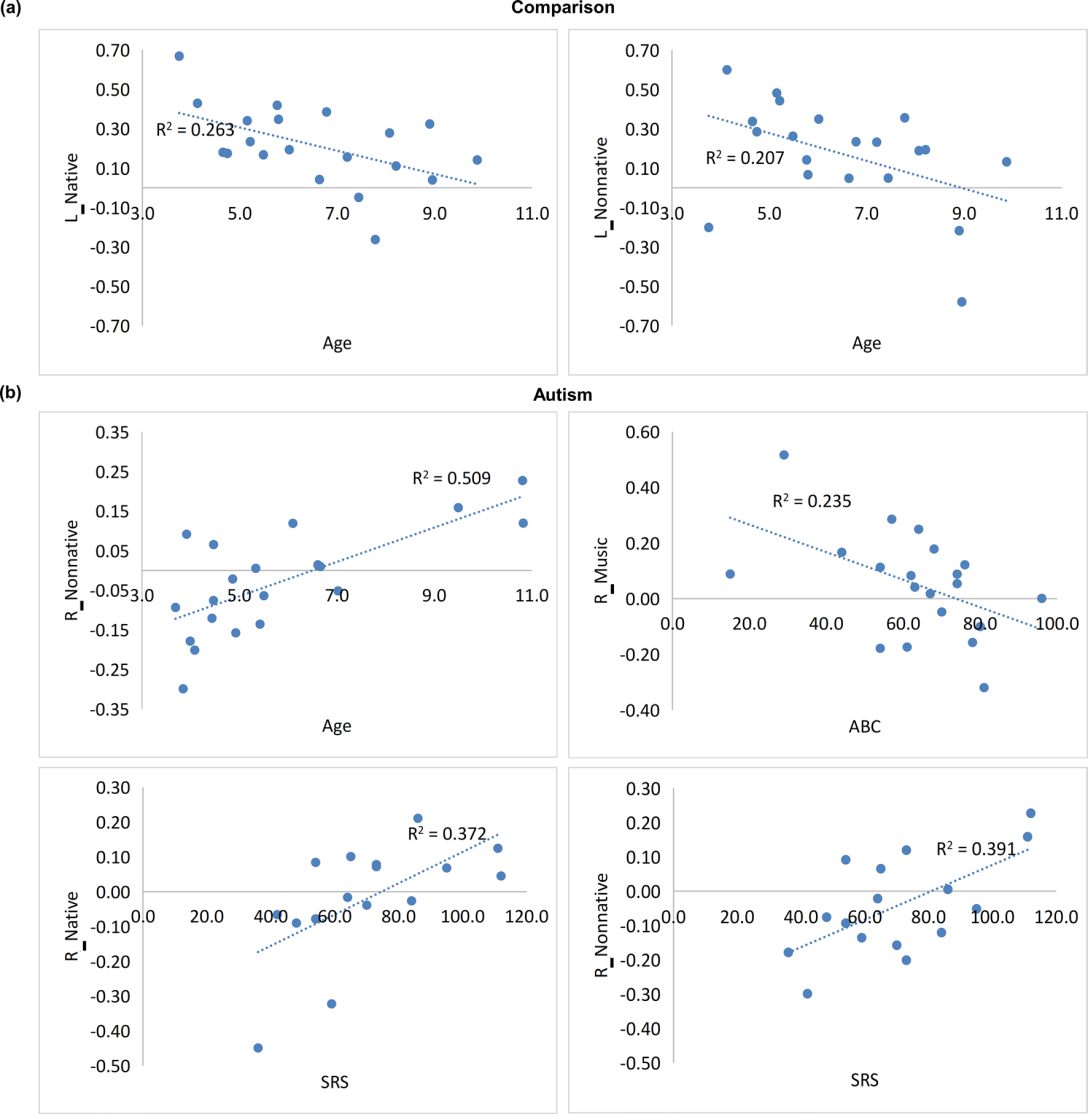
Scatter plots of significant brain-behavior correlations in the autism group and the comparison group. SRS: Social Response Scale. ABC: Autism Behavior Checklist. L = Left hemisphere; R = Right hemisphere.

In the comparison group, age was negatively correlated with the response to native and nonnative speech in the left hemisphere (*r*_native_ =  − 0.51, *r*_nonnative_ =  − 0.46, *p*s < 0.05, Fig. 6), displaying a developmental decrease of left hemisphere activity to native language.

In the autism group, several significant correlations were identified (Fig. 6). Specifically, response to nonnative speech in the right hemisphere increased with age (*r* = 0.71, *p* < 0.001). The SRS score was positively correlated right hemisphere response to native and nonnative speech (*r*_native_ = 0.61, *r*_nonnative_ = 0.63, *p*s < 0.05), suggesting a link between greater social symptoms and higher right hemisphere responsivity to speech. The ABC score was negatively correlated with the response of right hemisphere to music (*r* =  − 0.49, *p* < 0.05).

No other brain-behavior correlations were found (for a summary of the statistical results, see Supplementary Material Tables S3).

## Discussion

In this fNIRS study, we examined the activation patterns of frontal–temporal language regions during the processing of auditory stimuli with varying linguistic relevance in autistic children and nonautistic children. As hypothesized, the comparison group demonstrated an increasing left lateralization as the linguistic hierarchy of the auditory stimuli became more pronounced. In contrast, the autism group in the current study displayed a distinct lateralization pattern, showing heightened activity in the right hemisphere specifically when listening to scrambled native speech. These findings suggest that atypical language specialization in autism is associated with the phonological aspects of speech processing, and the right hemisphere involvement in autistic children may be influenced by sublexical information.

### Reduced neural specialization for language processing in autistic children

One main finding was the absence of overall lateralization for sound processing in the autism group as compared with the comparison group. Specifically, the comparison group showed a clear leftward asymmetry in the temporal lobe, whereas no such pattern was found in the autism group. Further analysis confirmed that the leftward asymmetry in the comparison group was unique to speech processing, in particular native speech. The robust left lateralization for native speech in the comparison group aligns well with a previous report of reliable left lateralization for language in the temporal network by the age of 7 years^26^.

Rather than finding focal cortical activity in terms of left lateralization, we observed more broadly activated cortical areas for sound processing in the autistic children compared to the nonautistic children. The significant cluster in response to normal native speech in the autism group was larger than that in the comparison group. In addition, the significant cluster in the autism group were located at supramarginal gyrus in the left hemisphere, whereas the comparison group’s significant cluster was concentrated in the Wernicke's area. This observation aligns with a recent meta-analysis of 11 studies utilizing fMRI^89^. The results of the meta-analysis showed that autistic individuals recruited additional areas in the middle temporal gyrus (MTG) and superior temporal gyrus (STG) during semantic processing, which were not activated in the TD brain. These findings, combined with the current results, suggest that these autistic children might need more widely distributed cortical resources for speech processing when compared to nonautistic children.

Moreover, we observed a positive association between right hemisphere response to nonnative speech and age in the autism group. This finding coincides with existing developmental data from autistic toddlers aged 12–48 months obtained using fMRI, which demonstrated an age-dependent increment of right lateralization for speech processing^29,42^. However, in the current study, age-related change in right hemisphere response was evident for nonnative speech only, but not for native speech and music. Because the nonnative speech is phonetic in nature but lacking linguistic relevance, we could imply that the developmental change was related to language-independent auditory function. Given that brain response to nonnative speech is considered an indicator of atypical neural commitment for language^90,91^, we have reasons to argue that an over-responsive right hemisphere for auditory processing might negatively impact neural commitment or specialization for linguistic processing in autistic children.

### Atypical right hemisphere involvement for sublexical speech processing in autistic children

The comparison group demonstrated a clear pattern of left lateralization in response to native speech, regardless of its semantic integrity. However, the left lateralization was only weakly present in the nonnative speech condition and was absent for music. This parametrically decreasing left-lateralization as a function of linguistic relevance fits well with our hypothesis about the nonautistic children. Notably, the lateralization measure but not the unilateral activation measure was uniquely reflective of linguistic processing. This result could signify a neurobehavioral outcome of language acquisition in which the brain becomes specialized for auditory signals of linguistic relevance. However, the systematic pattern of brain lateralization was absent in the autism group.

Another main finding was the lack of increasing left lateralization as a function of linguistic relevance in the autism group. As in the comparison group, the autism group showed leftward asymmetry when the speech was native and naturally presented; however, the asymmetry disappeared for the scrambled native speech associated with elevated responsivity in the right hemisphere. Lack of hemispheric asymmetry for speech processing is a highly replicated phenomenon in autism research^11–13,44,92^. Yet little is known about the specific component of language that could drive such differential lateralization in autistic children. The current findings provide important evidence of underlying correlates in this regard.

First, we consider psychoacoustic factors associated with the hyper-responsivity of the right hemisphere. Autistic individuals show an auditory bias for right-hemisphere functions such as the bias for spectral information^13,44,93^. This bias could be responsible for an atypical functional specialization for speech characterized by prominent pitch variations. In the current study, this type of speech was presented as scrambled Mandarin Chinese. The Chinese language is a typical tonal language with syllable-level pitch variations, a quality not present in the Nonnative Russian speech. Moreover, the Chinese speech in the Native-scramble condition did not have the grammatical markers that are present in naturally produced speech with regular word order. Thus, this scrambled speech might have appeared extra prosodic compared to normal Chinese. The right hemisphere of the Chinese autistic children might overly respond to these stimuli due to their spectrally prominent prosodic variations^33,43^. In line with this interpretation, our data demonstrated a trend of right lateralization in the autism group when listening to music, consistent with a right-hemisphere bias for melodic information.

Evidence of a second factor potentially responsible for the atypical lateralization concerns the “holistic” nature of processes carried out by the right hemisphere. A recent ERP study with school-age autistic children identified an atypical engagement of the right hemisphere in response to speech differing in linguistic relevance, namely, native versus nonnative pseudowords^43^. This finding suggests a sublexical component driving atypical cerebral specialization for language that is consistent with the current observation. This study did not support a pure auditory explanation because there was no difference between groups in lateralization for nonspeech sounds (native vs. nonnative prosodic acoustics). Instead, the researchers proposed a neurolinguistic explanation. Specifically, the right hemisphere is predominantly responsible for processing slow-changing holistic speech patterns such as supra-segmental word forms and envelope modulations^15,94–96^. Thus, it is conceivable that the atypical right hemisphere effect in autism could reflect overly holistic processing of speech. In other words, neurotypical children might hear the scrambled Chinese as combinations of fine-grained phonological units or potential words, whereas some autistic children might hear it as holistic speech forms^97^. Over-processing of such information might hinder their mastery of the correct phonological rules and grammatical structures. The over-responsivity of the right hemisphere might also inhibit paralinguistic and contextual processing functions that typically rely on the right hemisphere^29,40^.

A third factor that potentially contributes to the observed differential lateralization in autism could be restricted language experience. Although school-age autistic children have received ample language exposure, exposure is not equivalent to vocabulary and language experience. According to models of language neurodevelopment, brain functional change has its dynamic interplay with individual language experience^14,98–100^. Specifically, it was proposed that cerebral specialization for language is not only age-dependent but also synergistic with the mastery of the native phonology that facilitates efficient speech processing and learning^14^. In the current study, the autistic children displayed a bilateral activation pattern unique to the sublexical, phonological level of native speech processing associated with elevated right-hemisphere response, which could reflect an unreliable phonological representation insufficient to support a specialized language network. In line with this notion is the limited but converging evidence showing increased functional connectivity within homologue right-hemisphere regions of the core language network throughout development in autism^101^. An insufficiently specialized neural network could, in turn, impede efficient word use and acquisition, further limiting the child’s experience with language^14,100^.

### Limitations

First, we did not have standardized assessments for high-order receptive language such as syntactic ability or expressive language, which limits our ability to further examine the relationship between brain lateralization and language development in autism. For instance, previous work demonstrated a link between lateralization (as indexed by handedness) and expressive language, but not receptive language in autism^102^. PPVT as a measure of receptive vocabulary also has its apparent limitations in revealing structural language differences in children^103^. Second, the age range of our participants spanned across preschool to school age. We statistically controlled for age effects in the regression models, but it will also be useful to test the hypotheses in different age groups. Nonetheless, fNIRS data on language lateralization in preschoolers, and in children on the autism spectrum is extremely rare^104^. Finally, our speech materials were recorded by human voices rather than computer-generated speech. This method provides high ecological validity but lacks strict control over some acoustic parameters such as envelope, amplitude and frequency. However, the psychoacoustic differences across conditions were inherent in presenting stimuli that varied in linguistic relevance, and thus it might be impossible to fully eliminate the effects of these differences. Future investigations of fine-grained auditory and linguistic components are needed to better understand the underlying correlates of brain lateralization in autism.

## Conclusion

This study demonstrated the atypical brain lateralization in autistic children during speech processing. The nonautistic children showed an overall left-lateralization for sound processing, whereas the autism group displayed a bilateral activation pattern. Moreover, the nonautistic children's degree of left lateralization systematically decreased with decreasing linguistic relevance of the auditory stimuli. In contrast, the autism group’s brain lateralization did not follow the linguistic hierarchy. Instead, autistic children showed a lack of neural specialization for language at the sublexical level, driven by an over-responsivity of the right hemisphere. The findings underscore the potential auditory and neurolinguistic correlates of autism-related biases for right-hemisphere functions, and the role of language experiences in strengthening specialized language networks in the brain. However, it is essential to apply caution in interpreting these conclusions and acknowledge the limitations of generalizability across the entire spectrum, given the marked heterogeneity present in the language profiles of autistic individuals^2,3^. The ongoing debate surrounding the nature of language disorder as either a dimensional feature intrinsic to autism or a distinct condition additive to the autistic phenotype further underscores the complexity of the heterogeneity issue^2,3,105–107^. Future research may incorporate these considerations into study designs to advance our understanding of the underlying language mechanisms in autism.

### Supplementary Information

Supplementary Information.

## Data Availability

The datasets used and/or analyzed during the current study are available from the corresponding author on reasonable request.
