# Supplementary material for: Atypical brain lateralization for speech processing at the sublexical level in autistic children revealed by fNIRS
Source: Sci Rep. 2024 Feb 2;14:2776. doi: 10.1038/s41598-024-53128-7 (PMC10837203; doi:10.1038/s41598-024-53128-7)
Supplement: Supplementary file 1 — Supplementary Information. [file 41598_2024_53128_MOESM1_ESM.docx]

**Supplementary Results**

1. **Participant attrition details**

Table S1. Reasons for participant attrition in the autism group and the comparison group

| Group | Reasons for exclusion | | |
| --- | --- | --- | --- |
|  | Screening | Incompletion | Poor fNIRS signal: insufficient channels or trials after preprocessing |
| Autism | - | Refusing to wear fNIRS headband (*n* = 1) | Excessive movement and/or talking during test (*n* = 7); extra dense hair (n =2) |
| Nonautistic comparison | Not right-handed (*n* =1) | Crying (*n* = 1) | Extra dense hair (*n* =3) |

**1. Channels of significant activation identified by cluster-based permutation tests**

Figure S1. Channels showing significant HbO responses in the autism group

Figure S2. Channels showing significant HbO responses in the nonautistic comparison group

Figure S3. Channels showing significant HbO responses in the autism group but not in the comparison group

Table S2. Comparisons of significant clusters between groups

| Contrast | Index | Condition | Hemis | Channels  of cluster | Time (s) | *p* | *T-statistic* |
| --- | --- | --- | --- | --- | --- | --- | --- |
| Autism vs. comparison | HbO | Native-  scramble | Right | 25, 34, 35, 36, 44, 45, 46 | 5.0-13.5 | .007 * | 967.67 |
|  |  |  | Left | 17, 18, 28, 39, 48, 49 | 5.0-11.8 | .011 * | 687.09 |
|  | HbR | Native | Right | 3, 14, 24 | 20.3-21.9 | .078 * | -114.60 |
|  |  | Nonnative | Left | 28, 38, 39, 48, 50 | 5.0-13.4 | .014 * | 751.30 |

*Note.* The results were based on 1,000 permutations between stimulus condition and baseline (value = 0). Hemis = Hemisphere, HbO = oxyhemoglobin, HbR = deoxyhemoglobin. Two-sided test, **p* < .025. A negative T-statistic value indicates greater activation in the nonautistic comparison group than in the autism group.

**2. Hemisphere×Group interaction in each stimulus condition**

Analyses of Variance were conducted for each condition separately. Among the four conditions, the Native-scramble speech was the only condition showing significant Hemisphere × Group interaction (*F_(1, 38)_*=6.30, *p*=.016, *η*^2^_p_=.02). Left hemisphere activation was significantly greater than right hemisphere in the comparison group (*t_(1,38)_*=3.28, *p*=.002, *Cohen’s d*=1.04, 95% *CI*=[0.37, 1.69]) but not in the autism group (*t_(1,38)_*=-0.27, *p*=.789, *Cohen’s d*=0.09, 95% *CI*=[-0.70, 0.54]). Moreover, the comparison group had marginally stronger activation than the autism group in the left hemisphere (*t_(1,70)_*=1.93, *p*=.058, *Cohen’s d*=0.61, 95% *CI*=[-0.03, 1.24]) but not in the right hemisphere (*t_(1,70)_*=1.14, *p*=.258, *Cohen’s d*=0.36, 95% *CI*=[-0.27, 0.98]).

The main effect hemisphere was significant in the Native condition (*F_(1, 75)_*=16.96, *p<*.001, *η*^2^_p_=.18) and the Nonnative condition (*F_(1, 38)_*=7.31, *p=*.010, *η*^2^_p_=.08). The activation of the left hemisphere was stronger than that of the right hemisphere in both groups. There was no observable effects of group or hemisphere or interaction in the music condition.


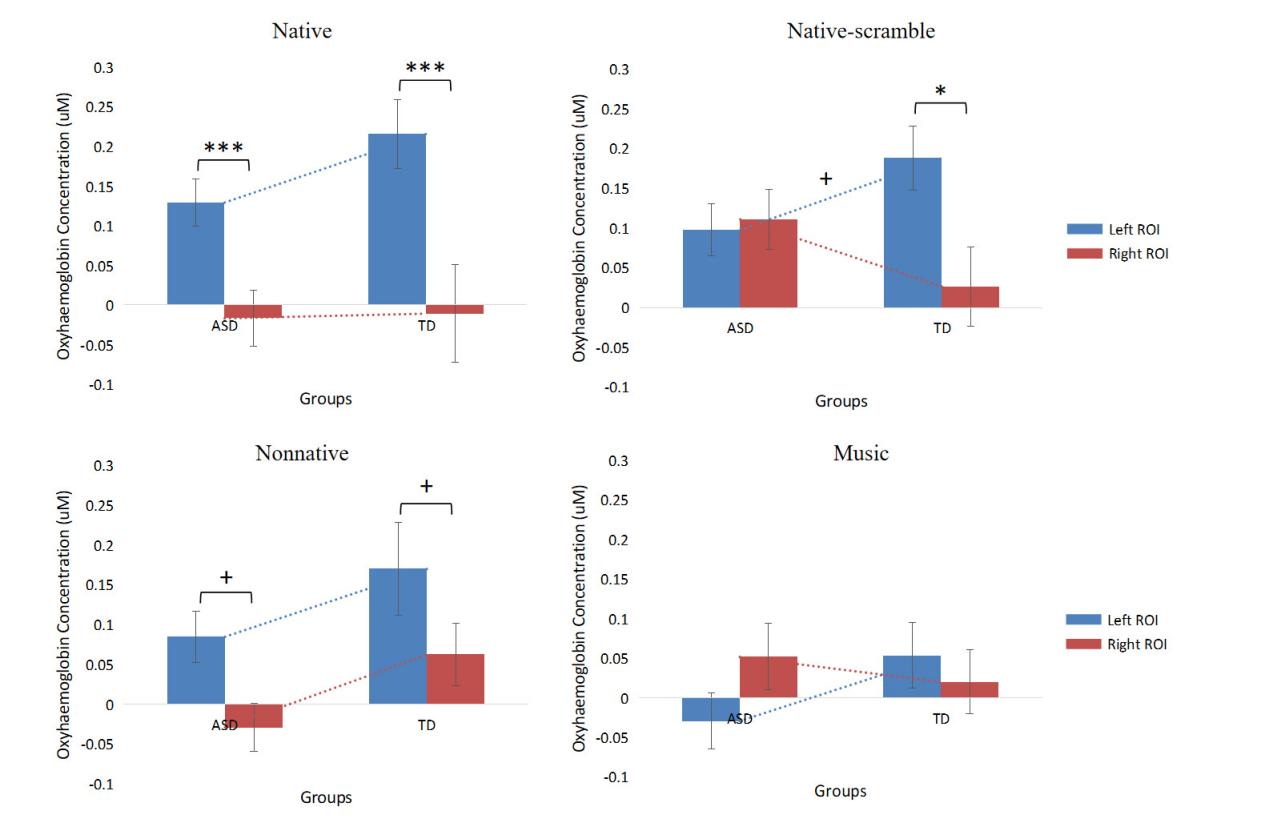


Figure S4. Hemisphere×Group interactions.

*Note.* ASD = the autism group. TD = the comparison group. Error bars indicate standard error. **p*<.05, ***p*<.01, ****p*<.001, +*p* <.1.

**Correlation results**

The tables present correlations among the brain and behavioral measures that were significant in *either* the autism group or the comparison group.

Table S3. Correlations among behavioral measures and hemisphere activation in the two groups

|  |  | Age | | PPVT-R | | SRS | | CARS | ABC |
| --- | --- | --- | --- | --- | --- | --- | --- | --- | --- |
|  |  | ASD | Comparison | ASD | Comparison | ASD | Comparison | ASD | ASD |
| PPVT-R | *r* | -0.41 | -0.40 | 1 | 1 | -0.41 | -0.12 | -0.42 | -0.12 |
|  | *n* | 14 | 18 | 14 | 18 | 12 | 12 | 14 | 14 |
| SRS | *r* | .72** | -0.36 | -0.41 | -0.12 | 1 | 1 | -0.10 | -0.06 |
|  | *n* | 16 | 12 | 12 | 12 | 16 | 12 | 16 | 16 |
| L_Native | *r* | -0.01 | -.51* | -0.17 | 0.34 | 0.06 | -0.24 | 0.18 | 0.36 |
|  | *n* | 20 | 20 | 14 | 18 | 16 | 12 | 20 | 20 |
| L_Nonnative | *r* | -0.09 | -.46* | 0.13 | 0.17 | -0.06 | 0.50 | 0.27 | 0.00 |
|  | *n* | 20 | 20 | 14 | 18 | 16 | 12 | 20 | 20 |
| R_Native | *r* | 0.30 | -0.14 | -0.22 | -0.19 | .61* | 0.35 | -0.01 | 0.29 |
|  | *n* | 20 | 20 | 14 | 18 | 16 | 12 | 20 | 20 |
| R_Nonnative | *r* | .71** | 0.06 | -0.20 | -0.41 | .63** | 0.15 | -0.07 | -0.02 |
|  | *n* | 20 | 20 | 14 | 18 | 16 | 12 | 20 | 20 |
| R_Music | *r* | -0.18 | -0.27 | 0.16 | 0.08 | -0.34 | 0.40 | 0.11 | -.49* |
|  | *n* | 20 | 20 | 14 | 18 | 16 | 12 | 20 | 20 |

*Note. n* = The number of participants who completed the scales; *r =* Pearson correlation coefficient. **p* < .05, ***p* < .01. L = Left hemisphere; R = Right hemisphere; PPVT-R: Age-based standards scores of Peabody Picture Vocabulary Test-Revised; SRS: Social Response Scale.
